# Valerian: No Evidence for Clinically Relevant Interactions

**DOI:** 10.1155/2014/879396

**Published:** 2014-06-30

**Authors:** Olaf Kelber, Karen Nieber, Karin Kraft

**Affiliations:** ^1^Working Group Efficacy, Safety and Interactions of Kooperation Phytopharmaka GbR, Plittersdorfer Straße 218, 53173 Bonn, Germany; ^2^Medical Information and Clinical Research, Steigerwald Arzneimittelwerk GmbH, 64295 Darmstadt, Germany; ^3^Institute of Pharmacy, University of Leipzig, 04103 Leipzig, Germany; ^4^Center of Internal Medicine, Universitätsmedizin Rostock, 18057 Rostock, Germany

## Abstract

In recent popular publications as well as in widely used information websites directed to cancer patients, valerian is claimed to have a potential of adverse interactions with anticancer drugs. This questions its use as a safe replacement for, for example, benzodiazepines. A review on the interaction potential of preparations from valerian root (*Valeriana officinalis* L. root) was therefore conducted. A data base search and search in a clinical drug interaction data base were conducted. Thereafter, a systematic assessment of publications was performed. Seven *in vitro* studies on six CYP 450 isoenzymes, on p-glycoprotein, and on two UGT isoenzymes were identified. However, the methodological assessment of these studies did not support their suitability for the prediction of clinically relevant interactions. In addition, clinical studies on various valerian preparations did not reveal any relevant interaction potential concerning CYP 1A2, 2D6, 2E1, and 3A4. Available animal and human pharmacodynamic studies did not verify any interaction potential. The interaction potential of valerian preparations therefore seems to be low and thereby without clinical relevance. We conclude that there is no specific evidence questioning their safety, also in cancer patients.

## 1. Introduction

Valerian “can interfere in an unwanted way with the oncological cancer therapy” or valerian “can diminish the efficacy of cancer therapeutics.” Statements like these can be found in published features on complementary medicine for cancer patients (Sloan Kettering Center 2012 [1]). In a very popular book on integrative oncology published by Sparreboom and Baker [2], it is stated that it is known for some herbal drugs that they can influence the activity of the cytochrome system and thereby may change the effect of chemotherapy. It is further stated that cancer patients often have difficulties to sleep and are restless, so that they can use herbal drugs such as valerian in order to avoid chemically defined sedatives and hypnotics as they fear becoming addicted. However, they state that warnings that valerian (*Valeriana officinalis*) may stimulate CYP 3A4.

This would reduce the effect of several cytostatic substances. For tamoxifen, the plasma level is reduced by CYP3A4 inductors, while inhibitors of CYP2D6 can lower the level of active metabolites, both leading to reduced efficacy. In case of cyclophosphamide, metabolic activation by CYP2D6 is necessary that is increased by inductors of this isoenzyme. The plasma level of several other cytostatic substances is lowered by CYP3A4 inductors. Examples are the epipodophyllotoxin derivative teniposide, camptothecin, tyrosine kinase inhibitors like imatinib, taxanes like paclitaxel and docetaxel, and vinca alkaloids like vincristine. CYP3A4 inductors also may reduce the activity of alkylating substances like ifosfamide and some antitumor antibiotics [2].

In several websites directed to patients (http://www.cancer.org, http://www.mskcc.org), the use of valerian in cancer is also questioned due to its interaction potential. By the way, Scientific American elected the latter website just a year after foundation as one of the best five US medicinal websites.

More than 70% of all cancer patients are very interested in herbal drugs. However, many of them do not inform their oncologist about their use [3, 4]. As 19 to 75% of all cancer patients suffer from sleep disturbances [5], warnings against the use of valerian preparations have a high impact on these patients. However, it has to be ascertained that warnings that valerian may cause interactions with other medicines are backed by valid evidence supporting such an assumption and that they are therefore scientifically correct. Therefore, we conducted a review in order to assess the interaction potential of valerian.

## 2. Efficacy and Safety of Valerian Extracts 

The clinical relevance of acute as well as chronic sleep disorders is obvious: epidemiological data show that they affect approximately one-third of the adult population [6, 7]. Treatment is indicated in about 15% of these cases [8, 9]. Insomnia, defined as insufficient quantity or quality of sleep resulting in compromised daytime alertness and activity, is a common condition. It can result in serious adverse consequences, including attention and memory impairment, depression, falls, and perceived reduced quality of life [10].

To the most common treatments of insomnia belong drugs [11], this however bears some problems. Benzodiazepines and imidazopyridines offer only short-term relief, while data on their long-term efficacy are scarce. Both drug classes have significant adverse effects such as serious psychomotor symptoms, behavioral aberrations, memory impairment resulting in injuries, respiratory depression, rebound insomnia, and paradoxical agitation. Especially for benzodiazepines, the potential for abuse is high [10]. Therefore, the National Institutes of Health consensus conference strongly discouraged chronic treatment of insomnia with benzodiazepines already in 2005 [11].

Sedating antihistamines, such as diphenhydramine, the active ingredient in most over-the-counter sleep aids, are associated with cognitive impairment, daytime drowsiness, and anticholinergic effects. There is no evidence-based data available on their efficacy improving insomnia or prolonging sleep. It therefore was recommended that they should be avoided in the elderly [10, 12]. Finally, antidepressants used for treating insomnia, such as trazodone, can produce dangerous and life-threatening adverse events due to their anticholinergic, cardiovascular, and neurologic actions [10, 12].

Herbal substances improving insomnia, such as valerian, hops, or passion flower, are well-known sleep aids. While marketed as food in the US, they are authorized or registered as medicines in Europe and many other regions, being mostly used in self-medication and holding widespread appeal, presumably because of their lower cost and higher range of safety when compared to chemically defined pharmaceuticals [13]. Among these, the roots of valerian (*Valeriana officinalis* L.) are the most familiar ones, especially in Europe. They improve the subjective experience of sleep when taken in the evening over a period of one or two weeks [14]. The constituents of valerian root include, among others, valepotriates (iridoids) and volatile oil, including monoterpenes and sesquiterpenes (valerenic acids). Commercially available extracts are free from valepotriates [15]. Recommended daily doses of valerian root extracts are about 600 mg, usually taken as capsules or tablets.

Several controlled clinical trials with various valerian extracts are available, and also a meta-analysis on eighteen randomized placebo-controlled trails was published. Its qualitative results suggest that valerian would be effective for a subjective improvement of insomnia, although its effectiveness could not be demonstrated with quantitative or objective measurements [16]. In a study conducted in cancer patients, an improvement in the primary variable, a sleep quality index based mainly on objective parameters, could not be demonstrated; however, fatigue and sleep problems were significantly improved [17].

The clinical studies available show an excellent short-term tolerability, and from several decades of clinical use within the frame of pharmacovigilance systems no data questioning its long-term safety have evolved, while prospective data are missing [18]. In contrast to classical sedatives, valerian extracts did not impair the ability to drive or to use machines, neither after single [19–22] nor after repeated doses [23]. Reports of putative adverse reactions are extremely rare and include one case of hepatic symptoms after prolonged treatment and one case of cardiac symptoms after discontinuation of a long-term treatment with very high doses, which were interpreted as a withdrawal reaction [24, 25]. In both case reports, outcome was benign, causality was questionable, and characteristics of the extract preparations were not provided.

While the side effect profile therefore is benign, a potential for adverse drug interactions has been claimed by some reviews [26], while other reviews did not [18, 27–32], so that a reevaluation of the existing evidence is necessary.

## 3. Material and Methods

For evaluation of the scientific data on the interaction potential of valerian (*Valeriana officinalis *L.), published data (experimental* in vitro* and* in vivo* studies, pharmacovigilance data from its long standing use as an authorized medicinal drug or supplement) were assessed and evaluated for identifying data on possible drug-herb interactions (Table 1). A search with the search terms (valerian or valeriana) was conducted in the databases MEDLINE and TOXLIT, via DIMDI (Cologne, Germany). All records, for which a relevance to the subject of herb-drug interactions of* Valeriana officinalis *L. could not be clearly excluded by the article title, were screened based on the abstracts. In all cases, where a relevance to the subject could thereby not be clearly ruled out, full-text articles were assessed and, as far as relevant, included into the review. Additionally MedIQ [33], a leading online database for the assessment of potential interactions in pharmacotherapy, was searched. Thereafter cross-referencing was conducted in order to identify and close potential gaps. Studies were assessed for completeness and validity of data on material and methods, on the potential clinical relevance of the results and on potential bias in the presentation of results and conclusions.

## 4. Results 

Eligible and included were all scientific publications on interactions of* Valeriana officinalis* L. root and preparations thereof. Among these are altogether 21 original publications. 11 of them present data from* in vitro* studies on pharmacokinetic interactions. One of these publications [34] contains, in addition, data on animal studies. Two publications [35, 37] present clinical studies on pharmacokinetic interactions. 8 studies are available on the subject of pharmacodynamic interactions, thereof two* in vitro* and three animal studies, one clinical study, and in addition two case reports.

### 4.1. Pharmacokinetic Interactions

Predicting pharmacokinetic herb-drug interactions is difficult because the pharmacological actions of the interacting drugs are often not related. This is also the case with the subject of this review, valerian as a treatment of insomnia, and chemically defined cytostatic therapies. Herb-drug interactions can occur on the levels of absorption, distribution, metabolism, or excretion and can change the amount and duration of the availability of a drug at the site of action. The interactions due to drug metabolism can be, as mentioned above, based on phase 1 metabolism (mainly involving cytochrome P 450 isoenzymes) or, rather rare, on phase 2 metabolism involving, for example, P-glycoproteins (P-gp), which is relevant for outward bound transport processes, for example, in the intestinal wall, or on conjugation, for example, with glucuronic acid [43]. In case that a specific herb-drug interaction is identified, its clinical significance depends on the degree of accumulation and the therapeutic window of the respective drug [44]. Also the dosage, time of administration, galenic properties, and coadministration as well as intrinsic and extrinsic factors may be of importance.

### 4.2. *In Vitro* Studies

Budzinski et al. [38] used a fluorometric assay for analysis of the* in vitro* CYP 3A4 inhibitory capability of dilutions of a valerian fluid extract (no further information available) using human CYP 3A4. An IC_50_ of 1.8% the undiluted extract in the reaction mixture was identified. The authors stated that “the* in vitro* interactions, though weak, may have clinical importance.” However, the valerian fluid extract tested was not specified, and the inhibitory concentration identified was very high. Additionally, the use of fluorometric methods is highly susceptible to interference by fluorescent herbal components [45]. Therefore, the results do not allow a valid extrapolation towards a clinically relevant effect.

Lefebvre et al. [39] determined the* in vitro *effects of 14 commercially available single entity and combination herbal products containing extracts of valerian root, on CYP 3A4-mediated metabolism and on P-gp transport. The extracts were prepared by extracting 100 mg of the powdered commercial preparations with 1 mL of water and 70% ethanol or acetonitrile and characterized by determination of total valerenic acids by HPLC. In a proportion of 1–5% of total assay volume, most extracts showed an inhibition to different extents. Six extracts had some inhibitory effects on P-gp. The authors concluded that “there is wide variation in commercially available samples of valerian root. The findings from this study suggest that valerian root may have an initial inhibitory effect, when taken with therapeutic products. Further work is warranted to determine whether valerian root can affect other CYP 450 isozymes and how the results of this* in vitro* investigation can be extrapolated to* in vivo* situations.” Indeed, several of the tested preparations are insufficiently characterized, as only the valerenic acid content is provided. In addition, only one rather high concentration of each extract was tested and, again, a fluorometric method was used, which is highly susceptible to interference by fluorescent herbal components. These limitations do not allow valid extrapolations with respect to a clinical relevance of the results.

The aim of a further study was to evaluate the* in vitro* effect of commercially available valerian medicinal products on the metabolic activities of the CYP 450 isoenzymes 1A2, 2D6, and 3A4. A valerian extract (drug extraction ratio 6 : 1, extraction solvent ethanol 60%) was incubated with human primary hepatocytes for three times within 48 h. The activities of the CYP isoenzymes were determined by analyzing the metabolites of test substances by HPLC [36, 46]. The herbal extract concentrations used in* in vitro* metabolic systems were claimed to cover the whole range of herbal concentrations occurring* in vivo*. Dose dependent and statistically significant increases in CYP 2D6 and CYP 3A4 were observed only with two concentrations (0.188 mg/mL and 1.875 mg/mL). The authors postulate an allosteric antagonism but also clearly point to the limitations of their study, as hepatocytes from only one donor were used. Therefore, the well-known polymorphisms in the CYP superfamily were not covered.

In a follow-up study, the same preparation was used, and an IC_50_ for CYP 3A4 inhibition at a concentration of 0.756 mg/mL was observed [40]. Slight but significant effects on bidirectional digoxin transport (involving P-gp) were found only with 1.875 mg/mL. The authors conclude that both CYP 3A4 and P-gp interactions are unlikely to be clinically relevant, as the systemic concentrations are probably much lower; therefore, respective IC_50_ values for P-gp cannot be reached* in vivo*. This conclusion is also applicable to their previous study [36] and to their study on CYP 2C19, which showed a weak induction by valerian root extracts [47].

In a further* in vitro *study conducted on mouse and human liver microsomes [34], the postulated inhibitory action of 2.5–75 *μ*g/mL of a valerian extract (aqueous ethanolic, not further defined) on CYP 1A1, CYP 1A2, CYP 2C, and CYP 3A was not confirmed.

In another study, the effect of valerian, valerian/hops extracts, and valerenic acid on the glucuronide conjugation of various substrates (17*α*-estradiol, acetaminophen, morphine, and testosterone) was determined using human liver microsomes [41]. Test substance was a valerian capsule (250 mg extract corresponding to 3.48 mg valerenic acid per capsule, further specification not available), which was extracted with 80% methanol (5 mL/capsule). Also, the activities of UGT 1A1 and UGT 2B7 were tested in the presence of 2.5 or 5.0 mL of valerian or valerian/hops extract per 250 mL final incubation volume. Valerenic acid significantly inhibited the glucuronidation by both microsomes and UGTs with the rather high concentration of 1 mg/mL. Due to this fact and the very high glucuronidation capacity of the liver, a clinical relevance of these results cannot be assumed.

Mohamed at al. [42] used an UGT 1A1 assay to test a commercially available valerian preparation (≥0.1% valerenic acid; extraction medium 70% ethanol, recommended daily dose 1000 mg) for inhibition of human estradiol-3-O-glucuronidation (E-3-G) in the same concentrations as Hellum et al. [36]. E-3-G was quantified by HPLC. IC_50_ was 0.562 mg/mL, which would be reached by dissolving one daily dose in a volume of 1.8 L. According to the authors, this volume is in the same order of magnitude as the volume of the intestine, and therefore a potential effect in the intestine could not be excluded [48]. However, even if a transient partial inhibition of UGT 1A1 in the intestine would occur, it would be transient, rather than a long-lasting enzyme induction, and could not to lead to a persistent change of bioavailability of another drug.

### 4.3. *In Vivo* Studies

In an* in vivo* study on mice, a valerian extract (aqueous ethanolic, not further described), given in a dose of 0.5% with the diet over 28 days (corresponding to a daily dose of 595 mg/kg in average) did neither affect the CYP content of the liver nor the activities of CYP 1A1, CYP 1A2, CYP 2C9, and CYP 3A4 [34].

In twelve healthy volunteers (6 males and 6 females, age 30.9 ± 7.2 years, nonsmokers from South Carolina), the effect of a valerian root extract on the activity of the drug-metabolizing enzymes CYP 2D6 and CYP 3A4 was tested [37]. Daily before going to bed, participants took two tablets, with 500 mg valerian extract each (extraction solvent ethanol 70%, valerenic acid content 5.51 mg/tablet), for 14 subsequent days. This dose is well within the range of recommended doses for valerian preparations. The probe drugs dextromethorphan (30 mg; CYP 2D6 activity) and alprazolam (2 mg; CYP 3A4 activity) were administered orally at baseline and after treatment with valerian, and dextromethorphan to dextrorphan metabolic ratios and alprazolam pharmacokinetics were determined. The ratio of dextromethorphan to dextrorphan increased slightly but significantly from 0.214 to 0.254. The maximum concentration of alprazolam was moderately increased for about 20% from 25 to 31 ng/mL (*P* < 0.05). This change is therefore within the frame of 80% to 125% rated as equivalent by FDA. Bioavailability of other medicines would therefore not be relevantly diminished and therapeutic efficacy of a therapy not questioned. Changes of other pharmacokinetic parameters were not detected. In conclusion valerian in therapeutic doses is unlikely to produce clinically relevant effects on CYP 2D6 and CYP 3A4 pathways which could diminish the therapeutic efficacy of other drugs.

In another study, twelve healthy volunteers (six men and six women, age mean ± SD = 24 ± 3 years, weight 69.3 ± 14.2 kg KGW) were randomly assigned to receive valerian (DER 4 : 1, no standardization claim) for 28 days, three times daily 125 mg [35]. Before and after the test period, the activities of CYP 3A4/5 (1-hydroxymidazolam/midazolam serum ratio), CYP 1A2 (paraxanthine/caffeine serum ratio), CYP 2E1 (hydroxychlorzoxazone/chlorzoxazone serum ratio), and CYP 2D6 (debrisoquine urinary recovery ratio) were determined. All subjects were nonsmokers and extensive metabolizers of CYP 2D6. No changes at all in phenotypic ratios were observed. The daily dose applied in this study was corresponding to 1.5 g drug, which is in the lower range of doses recommended, for example, in the monograph of the HMPC. Despite that, the fact that there was not even a tendency of an effect in the CYP isoenzymes tested underlines the assumption of the lack of an interaction potential in CYP enzymes.

### 4.4. Pharmacodynamic Interactions

Pharmacodynamic interactions include the concurrent administration of drugs having the same or opposing pharmacologic actions and also the change of the sensitivity or the responsiveness of the tissues to one drug, induced by another one. Many of these interactions can be predicted from knowledge of the pharmacology of each drug. They were proposed for valerian mainly with drugs influencing vigilance such as codeine, citalopram, and benzodiazepines. A presumable interaction with benzodiazepines, which are positive allosteric modulators on GABA-receptors, is based on* in vitro* data suggesting GABAergic mechanisms of action of valerian extracts [49, 50]. These studies however are inconclusive and require independent replication.

In rats an* in vivo* study was conducted on interactions between valerian root tincture (ethanol 100%, 1 : 10, not further characterized, daily therapeutic dose 3060 mg for an adult human) and haloperidol with respect to impaired liver or kidney functions. Valerian tincture was applied with the drinking water (1%, corresponding to an extract dose of 200–250 mg/kg bw/d). Haloperidol (38 mg/kg bw) was applied intramuscularly once every 4 weeks over 12 weeks beginning after 15 days of treatment with valerian [51]. While renal effects were lacking, in some of the parameters measured in liver homogenates, slight and statistically significant deviations from control values were observed, suggesting an additive effect of haloperidol and valerian, however of questionable relevance. Authors concluded that in humans a possible toxic additive effect would occur only at supratherapeutic doses. The same working group evaluated the effect of valerian in a rat model of orofacial dyskinesia using the same application procedure as described above. Valerian did not influence orofacial dyskinesia induced by haloperidol. Also oxidative stress parameters were not changed [52].

In another* in vivo* study on mice [53], 25 mg/kg of a valerian root dry extract was combined with 25 mg/kg of a liquorice extract (both prepared with ethanol 70%, not further characterized) or the benzodiazepine alprazolam (0.7 mg/kg). Tests were conducted in an elevated plus maze. Valerian and alprazolam, rather than liquorice, significantly increased time spent in the open arm, pointing to an anxiolytic effect. The effect of alprazolam, combined with liquorice or valerian, was significantly increased compared to each of the single substances. The authors discuss an improved bioavailability, for example, due to an increased gastrointestinal absorption induced by liquorice. The relevance in humans remains unclear.

In 48 healthy volunteers, pharmacodynamic interactions of single doses of valerian (100 mg/d, extract specification lacking) and propranolol (20 mg/d) were evaluated. The results were not presented in detail but indicated that the two drugs act independently from each other. Interactions with respect to heart rate and parameters of psychic strain could not be demonstrated [54].

A possible pharmacodynamic interaction of valerian preparations with other drugs is supported only by two case reports. A 40-year-old male patient had taken lorazepam (2 mg/d) for two month without side effects. Then he additionally took for two days an infusion of valerian root two hours before bedtime and, just before going to bed, an infusion of valerian root and passion flower herb (dose unknown) without side effects. On the third day he took, instead of the infusion, three tablets containing a combination of valerian root extract (300 mg) and passion flower root and herb extracts (350 mg; no further information available) before bedtime. He thereafter suffered from transient mild handshaking and drowsiness [55]. It was suspected that these symptoms were caused by an interaction between the herbal drugs and lorazepam, as they ceased after stopping herbal treatment. It is however questionable to ascribe the adverse event to valerian alone as the herbal preparation also contained a root and herb extract of passion flower, for which monographs are lacking. As the symptoms are also potential adverse effects of lorazepam itself, and no other similar cases have been described since then, and also a coincidence cannot be ruled out.

Another patient (39-year-old female) had taken for two month a daily dose of two tablets of a St. John's wort preparation and one tablet of a valerian preparation and additionally Loperamide [56]. After this time, she was hospitalized with a severe delirium. She claimed that the herbal treatment was a replacement for the opioid meperidine she had been taken before for reducing migraine. The authors proposed an interaction between the herbal drugs and Loperamide, involving MAO-inhibitory properties. Given that none of these herbal medicines has been proven to have MAO-inhibitory properties, while induction of delirium, and a positive drug screening on opioids was reported, the assumption of an involvement of the valerian preparation does not seem to be plausible.

## 5. Discussion

A good understanding of the mechanisms of drug-drug interactions is essential for assessing and minimizing clinical risks. Indeed many drug interactions are a result of inhibition or induction of CYP enzymes. This is especially true for many antineoplastic substances. However, interactions on P-glycoprotein and conjugation mechanisms should not be neglected. Additionally, pharmacodynamic interactions have to be considered.

Herbal medicinal drugs consist of multiple components. The complex nature of herbal drugs can provide broader information on multiple interaction mechanisms and the results may change due to environmental or manufacturing differences. More information is obtained than that with a single pure natural substance.

During the past decade, several potential mechanisms of interactions of valerian preparations, involving CYP enzymes, P-glycoprotein, and UGTs, have been studied* in vitro* [36, 38–42, 46]. Some of these studies pointed to a possible drug interaction potential by valerian extracts.However, critical assessment of these studies suggests that the clinical relevance of the findings is questionable due to various methodological limitations (Table 1). This is underlined by the available clinical interaction studies on CYP isoenzymes, which do not indicate a relevant drug interaction potential of valerian in healthy volunteers [35, 37].

In this context also the amiability of* in vitro* data to the situation in humans should be addressed. Especially the open questions of* in vivo* bioavailability and of the metabolism of extract components relevant for* in vitro* effects often prevent reliable extrapolations from* in vitro* to* in vivo* data. As* in vitro* and* in vivo* correlations usually are not available, results from* in vitro* interaction studies on herbal medical drugs may not simply be transferred to the* in vivo* situation. For valerian, and similarly also for *Echinacea*,* Ginkgo*, and hawthorn, clinically relevant interactions are lacking in* in vivo* studies, despite* in vitro* studies pointing to drug interactions* in vivo*. Since for herbal extracts a positive* in vitro and in vivo* correlation of data on drug bioavailability is rare, results from* in vitro* studies should be carefully interpreted [57].

Also the interpretation of metabolic studies in animals of different species should be critically evaluated, as often dosages far above those applied to humans are used and also regarding the enzyme variations in the species.

As for pharmacodynamic interactions of valerian, neither animal studies nor human data provide solid information for a possible risk [51, 52, 54]. Also the case reports are by no means convincing [55, 56]. At present therefore a relevant risk of pharmacodynamic interactions is not proven by valid clinical observations.

Altogether, this review could not identify studies showing a clinically relevant interaction effect of valerian. This confirms pharmacovigilance reviews of herbal medicinal products, which do not mention valerian at all [58–60] or claim valerian as safe [16, 27–31]. Block et al. [5] specifically pointed out that valerian is safe and efficient in patients undergoing cancer therapy. That therapeutic safety regarding interactions is high in valerian and is mentioned in the respective monograph of the HMPC [18] and also in a fact sheet of the Office of Dietary Supplements at the NIH [32]. In a data base for interactions of medicinal drugs (MedIQ), the rating “weak interaction” is given for CYP-isoenzymes, P-gp, and UGT, based on* in vitro* data. However, this rating is apparently the result of a merely formalistic approach and not the result from an analysis of the clinical relevance of these data.

## 6. Summary and Conclusions 

The use of valerian preparations is very common in patients with cancer, who often receive medicines with a narrow therapeutic window. It was claimed as not being safe with regards to interactions in earlier scientific reviews.

A critical analysis of interaction data and experimental setups in papers addressed to CYP 450 isoenzymes and P-gp as targets showed limited* in vitro* interactions. However, the relevance of herb-drug interactions becomes apparent only during clinical use. So the available studies on interactions on CYP 450 enzymes do not reveal clinically relevant interactions. Also the studies and other data on pharmacodynamic interactions do not support the assumption of clinically relevant interactions. Further adverse effects, if any, seem to be very rare and reversible.

In summary, it can be concluded that warnings regarding a specific risk of interactions of valerian are without any recent evidence. Instead, valerian turns out to be an advisable treatment option, as it has a more favorable safety profile than chemically defined hypnotics and as it also improves subjective parameters of sleep quality. Warnings directed to cancer patients, in whom restless nights are responsible for continued disruption of well-being and for further impairment of health, to abstain from its use, should therefore be substantiated and specifically take the comedication into account. Otherwise, they are likely to do more harm than good.

## Figures and Tables

**Table 1:** Potential pharmacokinetic interactions of valerian according to the published literature. The conclusions by the respective authors, the ratings given by MedIQ (http://www.mediq.ch, a Swiss independent interaction data base, which is structured according to mechanisms of interaction and includes herbal drugs), and the conclusions regarding the potential of clinically relevant interactions based on a critical analysis of the published studies are reported.

Metabolic pathway	*In vitro *studies	*In vivo *studies	Interaction potential (according to medIQ database [33])	Indication for a clinically relevant interaction
CYP 1A2	No effect [34]	No effect [35]		None

CYP 2D6	Induction [36]	No effect [35, 37]	Weak inhibitor	None

CYP 2E1		No effect [35]		None

CYP 3A4	Induction [36, 38]	No effect [34, 35, 37]	Weak inductor	None

P-glycoprotein	Weak inhibition? [39, 40]	No data available	Weak inhibitor	None

UGT 1A1	Weak inhibition? [41, 42]	No data available	Weak inhibitor	None

UGT 2B7	Weak inhibition? [41]	No data available	Weak inhibitor	None

CYP: cytochrome P450; UGT: uridine 5′-diphosphate glucuronosyltransferase.
